# Effect of toe exercises and toe grip strength on the treatment of primary metatarsalgia

**DOI:** 10.1186/s13018-020-02113-7

**Published:** 2020-12-02

**Authors:** Kentaro Amaha, Tatsuya Arimoto, Nobuto Kitamura

**Affiliations:** Department of Orthopedic Surgery, St Luke’s International Hospital, 9-1 Akashicho, Chuo-ku, Tokyo, 104-8560 Japan

**Keywords:** Toe exercise, Metatarsalgia, Conservative treatment, Toe grip strength, Chronic pain

## Abstract

**Background:**

The relationship of metatarsalgia and toe function is poorly understood. We investigated the efficacy of toe exercises for the treatment of metatarsalgia.

**Methods:**

Forty-one (56 feet) metatarsalgia patients (mean age ± SD: 63.4 ± 10.6) underwent toe strength measurement. We recorded pre- and post-treatment VAS score, AOFAS score, marble pickup, single-leg standing time (SLST), and compared in two subgroups to evaluate impact of disease duration on treatment outcome.

**Results:**

Post treatment, toe plantarflexion strength improved (all *p* < 0.01); VAS scores decreased (*p* < 0.01); AOFAS scores, marble pickup, and SLST improved (all *p* < 0.01). Patients symptomatic for > 1 year had significantly lower changes in VAS scores (*p* < 0.01). Multivariate analysis showed patients with longer disease duration, and larger body mass index had significantly lower improvement in VAS scores (*p =* 0.029 and *p* = 0.036, respectively). Device consistency assessed by ICC was excellent (0.89–0.97).

**Conclusion:**

Toe function and metatarsalgia are improved by toe exercises, suggesting that they are closely related.

## Introduction

Muscle mass decreases after approximately age 50 [1]. This age-related loss of muscle mass is termed “sarcopenia” and has been a focus of constant attention because of its association with mortality [2]. Lower ambulatory performance with aging is closely related to sarcopenia [3, 4]. In the lower extremities, the toes play a crucial role and assist stability during gait and balance tasks [5]. However, elderly patients tend to have decreased toe grip strength (TGS) of approximately 30%, compared to younger patients [6]. Loss of toe-muscle strength leads to impaired balance, thereby increasing the risk of falls [5]. Furthermore, toe grip weakness has been theorized to be associated with a range of forefoot deformities and disorders [7].

Metatarsalgia is one among the commonest conditions causing forefoot pain, characterized by pain in the front part of the foot, under the heads of the metatarsal bones. Metatarsalgia has various causes, including mechanical and iatrogenic factors [8]. Mechanical overload of the weightbearing structure is the fundamental etiology of primary metatarsalgia. Excessive forefoot loading is related to intrinsic abnormalities of metatarsal anatomy and gait mechanics [9]. With regard to gait mechanics, the toes play an important role in maintaining floor contact, which is shared with the metatarsal area during the toe-off phase in the gait cycle [10]. Therefore, the load on the forefoot is affected by the load on the toes. Thus, metatarsalgia seems to be closely related to the toe function.

There have been no reports of the relationship between toe function and primary metatarsalgia. However, toe function is difficult to evaluate. One of the methods to evaluate toe function is to measure the TGS. Despite a few reports on TGS measurement [6, 11], as yet, no fully validated study has assessed the absolute value of TGS in a clinical setting [12]. The lack of an evaluation method makes it difficult to understand complicated forefoot disorders. Horiuchi [13] developed a new device that measures TGS: a push-type toe-grip meter that can easily assess toe function.

This study was conducted to investigate TGS in metatarsalgia patients using the abovementioned novel device. Furthermore, we observed the effect of toe exercise on metatarsalgia to assess the relationship between TGS and clinical disease symptoms. We theorized that toe exercise can improve both TGS and clinical symptoms of metatarsalgia.

## Methods

### Patients

This single-center study was approved by the institutional review board (approval no. 17-R069) and conducted in accordance with the tenets of the Declaration of Helsinki. Between April 2012 and December 2015, all patients with metatarsalgia longer than 2 months without remission, regardless of treatment, were included in this study. Metatarsalgia was defined as weightbearing pain and/or tenderness on the plantar side of the lesser metatarsal head. Sixty patients were screened and examined according to the subject selection criteria by a single foot and ankle surgeon (KA) study investigator. As this was a retrospective case series, a statistically powered sample size was not calculated. Simple radiography of the patients’ feet was carried out, during weightbearing in the dorsoplantar view. The rate of patients’ complications of the hallux valgus, defined as a 20° or greater angle of the hallux valgus, was examined. Moreover, the forefoot length was assessed in the first metatarsal relative to the second metatarsal. Patients were divided into 3 groups according to this length: in the index minus, the first metatarsal was shorter than the second and the following metatarsals became progressively shorter; in index plus, the first metatarsal was larger than the second; and in index plus-minus, the first and second metatarsals were approximately the same length. Furthermore, all patients underwent MRI and were confirmed not to have rupture of plantar plates or Freiberg’s disease. The exclusion criteria were as follows: patients with conditions such as rigid forefoot deformities (e.g., hammer toes, claw toes), patients with infection, crystal arthritis, previous foot surgery, major trauma, rheumatic disease, and neurological disease (e.g., cerebral infarction, parkinsonism, radiculopathy, and Morton’s neuroma). After implementing the exclusion criteria, we included 56 feet (41 patients) older than age 20 who were not allowed to take medications or receive any insole treatment during the study period. All patients provided written informed consent.

### Toe exercise

Patients received an 8-week toe functional exercise program conducted under a physiotherapist’s guidance. We administered simple exercises such that elderly individuals could understand them. The patients sat in an upright position, placed a towel on the floor, and placed one foot on it, one shoulder-width apart. Then, they used their toes to scrunch up the towel, making sure to keep the rest of their foot in contact with the ground. Three sets of 15 scrunches were performed on each foot. In addition to the towel exercise, curling and spreading out of all toes was done. Patients were instructed to perform the exercises for 10 min 2 times a day for 2 months.

### Toe-grip strength meter

**Fig. 1:**
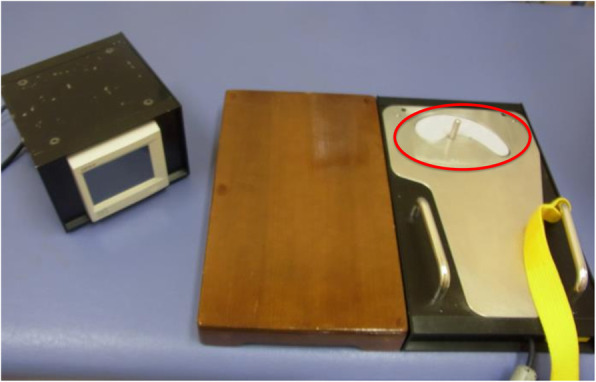
Push-type toe-grip strength meter. Toes press the floor, facing the strain gauge installed in the cantilever (red circle)

**Fig. 2:**
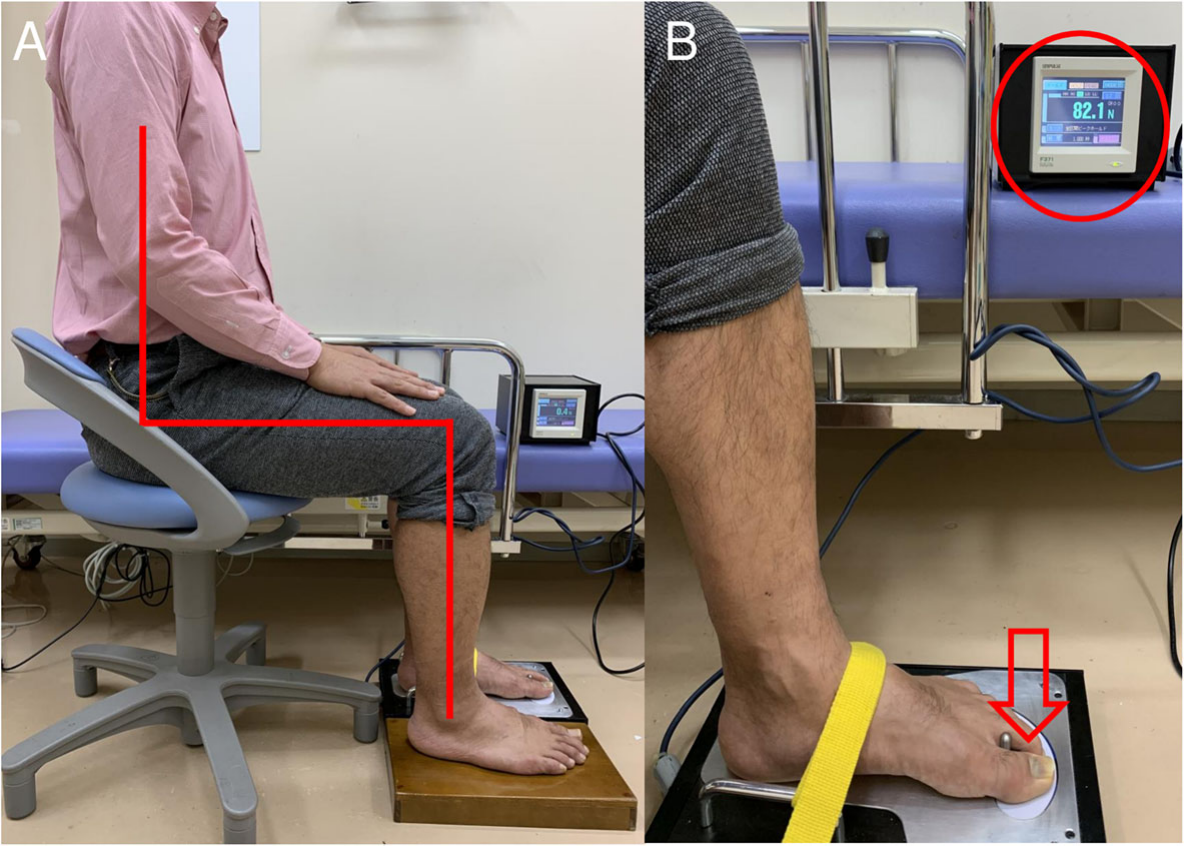
Measurement of TGS. **a** Measurement in the seated position, foot is placed on the positioning bar and strapped tightly to the device. The patients sat on a chair adjusted to their height with their knee and hip joints precisely at 90°. Then, the floor is pressed as firmly as possible with the foot. **b** The greatest value (Newton: N) while the toes are gripping (red arrow) is automatically recorded as the maximal peak force on the black box screen (red circle)

TGS was measured using the push-type toe-grip strength meter (Fig. 1) [13]. Using the strain gauge installed in the cantilever, the device measures the strength of the toes pressing the floor. For the measurement, a foot was placed on the positioning bar of the device and strapped tightly to restrict upward motion during measurement. The measurement was started after the confirmation of zero on the device’s screen. The second foot could be measured by turning the measurement board upside down. We measured all the TGS values before and after the exercise program in both upright and sitting positions. In the upright position, patients stood upright on the measurement board facing forward. Special care was taken not to have the patient’s head down, causing extra weight to be placed on the toes. During measurement in the seated position, patients sat on a chair adjusted to their height with their knee and hip joints precisely at 90°. Then, the patients gripped their toes using maximal force in both positions. The highest value (Newton: N) of force with which toes were gripped was automatically recorded as the maximal peak force on the device’s screen (Fig. 2). We undertook the measurement twice on the same day. The average of the two measured values was recorded.

### Assessment

Patients were also assessed by using the visual analog scale (VAS) for pain, the American Orthopedic Foot and Ankle Society (AOFAS) hallux metatarsophalangeal–interphalangeal scale (scale of 100 points), the number of marbles that the patient could pick up using their toes in 10 s while seated (picking up the marbles [PUM]) and observing the number of seconds the patient was able to stand on a single leg for up to 60 s (single-leg standing time [SLST]). The VAS, consisting of horizontal lines of a 100-mm length, was self-recorded. For pain intensity, the scale is anchored by “no pain” (scale of 0) and “worst imaginable pain” (scale of 100). To investigate whether the duration of disease affects the outcome of treatment, patients were divided into two groups based on disease durations of more than 1 year or less, and the average difference in the degree of improvement between the pre- and post-treatment measures was examined. Multivariate analysis was conducted to investigate which background factor correlated with the degree of improvement in the TGS in the upright and sitting positions, degree of improvement in AOFAS score, and improvements in VAS scores. The reliability of the push-type toe-grip strength meter was assessed with the Bland-Altman plot using the intra-rater correlation coefficient (ICC). During the measurement of TGS, the inter-rater reliability was not examined because the value is only read off of the screen of the device.

### Statistical analysis

To compare the pre-exercise (pre-ex) and post-exercise (post-ex) values in relation to the parameters evaluated, the paired *t* test for independent samples was used. The average difference in the degree of improvement between the pre- and post-treatment measures was also evaluated by the paired *t* test. A multivariate generalized linear model, using normal distribution, was used to investigate the relationship between the background factors, including age, sex, body mass index (BMI), duration of disease, affected side, forefoot morphology, and outcomes, including improvement of TGS in upright and seated positions, VAS scores, and AOFAS scores. In the affected-side analysis, the right and left feet were evaluated as a comparison based on bilateral feet measurements. In the forefoot morphology, plus and plus-minus feet were evaluated as a comparison based on minus feet. To determine the inter-rater reliability of the push-type toe-grip strength meter, the Bland–Altman plot was constructed to assess the agreement. Intra-class correlation coefficients for agreement were calculated. The data were analyzed by SPSS statistical software (version 7, Chicago, Illinois). The level of significance was set at *p <* 0.05.

## Results

Basic characteristics of the current study participants are shown in Table 1. In total, 56 feet with metatarsalgia, 32 on the right side and 24 on the left side, were included. Symptoms involved both feet in 14 patients. The average duration of metatarsalgia was 21.2 ± 33.2 (range 2–120) months. In the evaluation of forefoot morphology, index minus was present in two thirds of patients, and index plus was only present in 1 case. A total of 20 patients, 30.3% of whom had a hallux valgus angle of 20° or more on foot X-ray (Table 1).

**Table 1:** Basic characteristics

Variables	
Age (years)	63.4±10.6
Gender, male/female	7/34
Height (cm)	156.6±7.7
Weight (kg)	52.8±7.3
BMI (kg/m^2^)	21.5±2.5
Duration of metatarsalgia (months)	21.2±33.2
Affected side, left/right/bilateral	10/18/14
Hallux valgus (%)	17/56 (30.3%)
Morphology, minus/plus-minus/plus	38/17/1

Values are provided as mean ± standard deviation

Abbreviations: *BMI* body mass index

In the unilateral cohort, in the sitting position, the grip strength of the toe of the affected foot was significantly weaker at 23 ± 17.2 N compared to 28.8 ± 18 N on the unaffected side (*p* < 0.01). In the upright position, the strength of the affected foot was 51.8 ± 37.9, which was weaker than the strength of the unaffected foot at 57.8 ± 31.3 N, but this difference was not significant. After the 8-week exercise program, all variables, including toe plantar-flexion strength, significantly increased in both standing and seated positions. Table 2 shows the change in values. The mean VAS score significantly decreased from 5.2 to 2.5 (*p* = 0.00). The AOFAS score significantly improved from 67.2 to 77.1 (*p* = 0.00) and PUM also significantly improved from 7.7 to 9.5 (*p* = 0.00).

**Table 2:** Pre- and post-exercise values

	Pre-ex	Post-ex	*P*
Upright TGS (N)	54.4±36.1	67.4±37.9	0.01
Sitting TGS (N)	24.7±16.8	32.1±17.9	<0.01
AOFAS score	66.7±16	79.9±12.5	<0.01
VAS	4.4±1.6	1.8±1.4	<0.01
Marble pickup (number)	5.9±2.9	8.6±2.3	<0.01
SLST (sec)	39.4±23.2	49.3±19	<0.01

Values are provided as the mean±standard deviation

Abbreviations: *TGS* toe-grip strength, *ex* exercise, *AOFAS* American Orthopedic Foot and Ankle Society, *VAS* visual analog scale, *SLST* single-leg standing time

After dividing patients into two groups according to disease duration, there were 20 patients in the more than 1-year group and 36 in the less than 1-year group. With the available numbers, no significant difference in TGS or AOFAS scores could be detected pre- and post-exercise in neither upright nor sitting positions. Only VAS scores were significantly different between the two groups. Patients with symptoms persisting for more than 1 year showed a significantly lower change in VAS scores (Table 3). The multivariate generalized linear model showed that the plus-minus foot had significant improvement in AOFAS scores compared to that of the minus foot between the upright and sitting positions. Regarding VAS scores, improvement was worse in patients with a long history of disease and high BMI (Table 4). The results from the Bland–Altman plots indicated that there was excellent agreement among the positions, both on the affected side and the unaffected side (0.89–0.97). No adverse events were noted.

**Table 3:** Mean difference between pre- and post-operative measures in two groups of differing disease duration

Values	Over 1 year (*n* = 20)	Less than 1 year (*n* = 36)	*p*
Upright TGS	15.9±26.8	11.3±30.3	0.56
Sitting TGS	9.2±8.6	6.3±15.2	0.35
VAS	1.5±1.19	3±1.5	<0.01
AOFAS score	11.7±10.9	14.1±10.6	0.42

Values are provided as the mean±standard deviation

Abbreviations: *TGS* toe-grip strength, *ex* exercise, *AOFAS* American Orthopedic Foot and Ankle Society, *VAS* visual analog scale

**Table 4:** Multivariate generalized linear model for coefficients among the variables

Values		Age	Gender	BMI	Duration of disease	Right foot	Left foot	Plus-minus	Plus
Upright TGS change	Coef	− 0.09	11.17	− 2.04	− 0.09	2.48	− 14.9	4.87	2.17
	[95% Conf. Int]	[− 0.90, 0.718]	[− 11.50, 33.93]	[− 5.22, 1.14]	[− 0.34, 0.15]	[− 21.59, 6.55 ]	[− 37.43, 7.63]	[− 12.69, 22.42]	[− 56.16, 60.50]
	P value	0.827	0.336	0.208	0.437	0.84	0.195	0.587	0.942
Sitting TGS change	Coef	− 0.08	3.98	0.11	− 0.02	4.21	− 0.97	8.48	9.33
	[95% Conf. Int]	[− 0.46, 0.30]	[− 6.80, 14.76]	[− 1.40, 1.61]	[− 0.14, 0.09]	[− 7.18, 15.61]	[− 11.64, 9.70]	[0.17, 16.80]	[− 18.29, 36.96]
	*P* value	0.679	0.469	0.888	0.682	0.469	0.858	0.046*	0.508
VAS change	Coef	0.01	0.04	− 0.17	− 0.01	− 0.06	− 0.93	0.57	− 0.43
	[95% Conf. Int]	[− 0.32, 0.05]	[− 1.12, 1.21]	[− 0.33, − 0.01]	[− 0.027, − 0.01]	[− 1.30, 1.17]	[− 2.08, 0.23]	[− 0.33, 1.47]	[− 3.42, 2.56]
	*P* value	0.669	0.942	0.036*	0.029*	0.918	0.115	0.212	0.777
AOFAS change	Coef	0.06	− 2.71	− 0.23	<0.01	− 0.63	− 1.36	9.06	7.69
	[95% Conf. Int]	[− 0.24, 0.36]	[− 11.15, 5.74]	[− 1.41, 0.95]	[− 0.09, 0.10]	[− 9.56, 8.30]	[− 9.73, 7.00]	[2.54, 15.57]	[− 13.97, 29.34]
	*P* value	0.701	0.53	0.7	0.927	0.89	0.749	0.006*	0.487

Coeficient is significant at the 0.05 level

Abbreviations: *Coef* β coeficient, *95% Conf. Int* 95% confidence intervals, *TGS* toe-grip strength

## Discussion

The unique findings of this study provide objective evidence confirming the effects of toe exercise on metatarsalgia. The use of insoles was the standard treatment for metatarsalgia, and its effect was reported to improve VAS by an average of 1 point [14]. In this study, the average improvement was 2.7 points, and it resulted in less pain than do insoles. Previous reports have evaluated TGS between the sitting and upright positions [15]. They found that there was no difference in the TGS between the upright and sitting positions. Contrary to the previous report, TGS in the sitting position was significantly lower than that in the upright position. The push-type toe-grip strength meter in the current study measures the toes’ pressing force applied to the ground, whereas the pull-type meter used in the previous study measures the toes’ pulling force in the proximal direction, similar to the hand-grip dynamometer. As the shape of the toes is different for each person, it is questionable whether the pulling bar of the pull-type device fits all foot shapes. Furthermore, we placed emphasis on the force that presses or steps on the ground as a function of the toes. In that sense, we believe that the push-type device reflects the function of the toes more clearly.

The current study also demonstrated that the affected foot has significantly weaker TGS in the sitting position compared to that in the unaffected foot, but no significant difference was observed in the upright position. It is unclear why the difference in the upright case is not manifested; it may possibly be due to some compensatory function working in the upright position to maintain the TGS.

Primary metatarsalgia is considered an abnormality that is related to the anatomy of the metatarsal bones as well as to the relationship between metatarsal bones and the rest of the foot, which leads to overload [9]. Metatarsal bone-length discrepancy has received the most attention thus far [16]. The present study showed that patients with index-minus accounted for most of the participants, suggesting that the second metatarsal was relatively long, and the metatarsal length seemed to be involved in the onset of metatarsalgia. However, as the length of the metatarsal bone cannot be changed except for surgery, another viewpoint is required for conservative treatment.

During walking, the load increases to about 2 times that of body weight before toe-off at the MTP joints [17] due to the combined effect of forward-falling and ground reaction force loads applied to the forefoot. When the toes lose the ability to functionally push off the ground, it places an increased load on the metatarsal area. This repetitive overloading in the metatarsal area causes metatarsalgia.

We observed an improvement in PUM, indicating that exercise not only improves strength but also enhances toe function. Therefore, metatarsalgia may be relieved as a consequence of an improvement in toe function.

Patients with long durations of metatarsalgia (more than 1 year) did not have improved VAS scores to a great extent in this study. The multivariate generalized linear model showed that the foot with plus-minus morphology had a significant improvement in AOFAS scores compared to that in the foot with minus morphology in the upright and sitting positions. Regarding VAS scores, improvement was worse in patients with a long history of disease and high BMI. In terms of the long duration of metatarsalgia, chronic pain is generally considered difficult to cure. Although there is an opinion that it is difficult to create a temporal boundary in terms of the difference between acute and chronic pain, generally, pain lasting 12 weeks or more is regarded as chronic pain [18]. The cause of pain in metatarsalgia is unclear, but it is presumed that the acute phase involves nociceptive pain. The improvement in pain was worse in chronic cases, suggesting that mixed pain, involving nociceptive, nociplastic, and neuropathic pain, is involved in chronic disease [19]. For patients with hallux valgus, waiting for elective surgery has been associated with less improvement in physical function outcomes following surgery [20], which supports our findings. Our results suggest that a different treatment strategy may be needed for chronic pain in metatarsalgia lasting more than 1 year.

We found that an increased BMI positively correlated with worse improvement in VAS scores. Moreover, previous reports showed a high BMI was positively correlated with elevated opioid consumption rates [21]. However, this result may stem from doctors prescribing a high number of opioids because of the patient being obese. The relationship between obesity and pain is still unclear.

The strength of this study is in the assessment of the toes through the use of reproducible absolute values of TGS, showing that weak toes tend to promote metatarsalgia. This device measures TGS by measuring the strength used to press the toes into the floor. The force of toe plantar-flexion is the strength of sustaining one’s weight, shared with the ball area in the toe-off phase. Therefore, this movement directly reflects the power of the toe-off phase by pressing off the ground. This push-type toe-grip strength meter is a useful device to detect toe weakness in the clinical setting.

The current study has certain limitations. First, the participants were recruited from a single institution; hence, the results may not be generalizable. Second, we have no objective proof of overloading in the metatarsal area in people with weak toe grip. Proper walking not only needs a certain level of plantar-flexion strength but also the ability to move the toes in order to work effectively. The ability of toes to control the loading warrants further investigation. Third, pain may deteriorate due to the natural course of metatarsalgia progression. Since we did not include a control group, further investigations in the form of randomized controlled studies are required to investigate the effectiveness of toe exercises in metatarsalgia.

## Conclusion

We investigated TGS in metatarsalgia patients by using a new device. To our knowledge, this is the first observational study to determine the relationship between TGS and clinical symptoms. Our results provide objective evidence confirming the effect of toe exercises. Patients with metatarsalgia tend to have a weaker TGS. Regaining toe strength could ease the pain caused by metatarsalgia; however, patients with minus morphology feet are likely to develop metatarsalgia, resulting in difficulty in healing, and patients with chronic metatarsalgia for more than 1 year show a significantly lower improvement in VAS scores. The push-type toe-grip strength meter is highly reproducible and a clinically useful device.

## Data Availability

All the data will be available upon motivated request to the corresponding author of the present paper.
